# Differential Effects of Human L1CAM Mutations on Complementing Guidance and Synaptic Defects in *Drosophila melanogaster*

**DOI:** 10.1371/journal.pone.0076974

**Published:** 2013-10-14

**Authors:** Sirisha Kudumala, Julie Freund, Michael Hortsch, Tanja A. Godenschwege

**Affiliations:** 1 Department of Biological Sciences, Florida Atlantic University, John D MacArthur Campus, Jupiter, Florida, United States of America; 2 Department of Cell and Developmental Biology, University of Michigan, Ann Arbor, Michigan, United States of America; Trinity College Dublin, Ireland

## Abstract

A large number of different pathological L1CAM mutations have been identified that result in a broad spectrum of neurological and non-neurological phenotypes. While many of these mutations have been characterized for their effects on homophilic and heterophilic interactions, as well as expression levels in vitro, there are only few studies on their biological consequences in vivo. The single L1-type CAM gene in Drosophila, *neuroglian* (*nrg*), has distinct functions during axon guidance and synapse formation and the phenotypes of *nrg* mutants can be rescued by the expression of human L1CAM. We previously showed that the highly conserved intracellular FIGQY Ankyrin-binding motif is required for L1CAM-mediated synapse formation, but not for neurite outgrowth or axon guidance of the Drosophila giant fiber (GF) neuron. Here, we use the GF as a model neuron to characterize the pathogenic L120V, Y1070C, C264Y, H210Q, E309K and R184Q extracellular L1CAM missense mutations and a L1CAM protein with a disrupted ezrin–moesin–radixin (ERM) binding site to investigate the signaling requirements for neuronal development. We report that different L1CAM mutations have distinct effects on axon guidance and synapse formation. Furthermore, L1CAM homophilic binding and signaling via the ERM motif is essential for axon guidance in Drosophila. In addition, the human pathological H210Q, R184Q and Y1070C, but not the E309K and L120V L1CAM mutations affect outside-in signaling via the FIGQY Ankyrin binding domain which is required for synapse formation. Thus, the pathological phenotypes observed in humans are likely to be caused by the disruption of signaling required for both, guidance and synaptogenesis.

## Introduction

**Figure 1:**
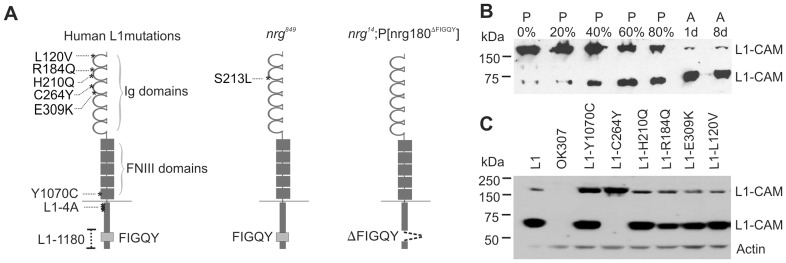
L1-type CAM mutations and their expression in the Drosophila nervous system. (A) Domain structure of L1CAM showing the locations (asterisks) of different pathological mutations. Mutations L120V, R184Q, H210Q, C264Y, and E309K are in the extracellular Immunoglobulin (Ig) like domains, while Y1070C is in the fifth Fibronectin domain. In the L1-4A protein four amino acids in juxtamembrane region (KGGKYSV) are replaced by alanine residues. The L1-1180 protein lacks part of its C-terminus including the FIGQY motif. The domain structure of Neuroglian (Nrg) indicates the site of the S213L mutation in the second Ig like domain of the *nrg^849^* protein. The genomic pacman P[nrg180^ΔFIGQY^] rescue construct in the *nrg^14^* null mutant background expresses neuronal Nrg^180^ that lacks the FIGQY motif. (B) Western blots of pupal stages (in %) and adult (1 and 8 days) flies expressing human UAS-human L1CAM with the OK307 Gal4-driver. Antibodies against the intracellular domain of L1CAM detected 200 kDa and 65 kDa bands. Proteolytic L1CAM cleavage increases with the maturation of the fly nervous system during pupal development and reaches its maximum in the adult. (C) Western blot of transgenic expression of wild-type and mutant UAS-L1 constructs in the wild-type background with the OK307 Gal-4 driver. With an antibody against the intracellular domain of L1CAM a 200 kDa band and 65 kDa band was detected for all L1CAM-constructs except for UAS-L1-C264Y. Only the full length 200 kDa L1CAM form was detected for this construct. No L1CAM protein was detectable in OK307 flies, which served as a negative control. Anti-actin labeling was used as a loading control.

The L1 family of cell adhesion molecules is well-known for its divergent roles during early nervous system development, specifically for axonal outgrowth, myelination, fasciculation, as well as long term potentiation, synapse formation and maintenance [1], [2], [3], [4], [5], [6]. The overall domain structure is not only conserved between the four vertebrate L1-type proteins (L1CAM, Neurofascin, NrCAM and CHL1), but also across most metazoan phyla [7], [8]. L1CAM is comprised of six Immunoglobulin (Ig)- and five Fibronectin (FNIII)-type extracellular domains. The cytoplasmic protein domain contains a highly conserved intracellular FIGQY motif, which interacts with Ankyrin proteins (Fig. 1A). More than 170 pathological mutations have been identified in the human L1CAM gene, which are associated with a broad spectrum of phenotypes. These include mostly neurological defects, such as mental retardation, spasticity and corpus callosum hypoplasia [9], [10], [11], [12], [13]. Several pathological L1 missense mutations have been studied in vitro, specifically in respect to homophilic and heterophilic interactions, cell surface expression and their effects on neurite outgrowth and axon branching in cell culture [13], [14], [15], [16], [17]. However, the biological in vivo consequences of these mutations at cellular level are less well studied. Various studies found reduced homophilic (H210Q, R184Q, C264Y) and/or heterophilic binding (R184Q), defects in transport (R184Q, W1036L), cell surface expression (C264Y, E309K, Y1070C) and reduced activation of EGFR signaling (E309K and Y1070C) [13], [14], [15], [16], [17].

In the central nervous system, the sole L1-type Drosophila *neuroglian* (*nrg*) gene generates two alternately spliced isoforms [18], [19]. Nrg^180^ is expressed exclusively in the nervous system and Nrg^167^ mostly in non neuronal tissue. Similar to vertebrates, Nrg plays distinct roles during axonal guidance and synapse formation [6], [20], [21]. The Ankyrin binding FIGQY motif and the remainder of intracellular domain in the Nrg^180^ and Nrg^167^ isoforms are encoded by distinct exons allowing the characterization of the role of the FIGQY motif for either isoform separately [19], [20]. We used the giant fiber (GF) circuit, which mediates the escape response in the fly [22], and the larval neuromuscular junction to characterize the role of Nrg and its FIGQY motif at a single cell level in vivo [20]. Using a pacman-based genomic rescue approach, we recently demonstrated that the highly conserved intracellular FIGQY motif of Nrg^180^ is required for synapse formation and synapse stability but not for axon growth and guidance in the GF circuit as well as at the larval neuromuscular junction [20]. In contrast, no phenotypes were seen in *nrg^14^*;P[nrg167^ΔFIGQY^] mutants in either model neuron.

The position of the S213L missense mutation in the second Ig domain of *nrg^849^* mutants is analogous to the pathological human L1-H210Q mutation [23]. Similar to human L1-H210Q, Nrg-S213L disrupts homophilic binding as well as causes learning and motor defects along with anatomical brain defects [23], [24], [25]. In the GF circuit, *nrg^849^* mutants exhibit axonal guidance as well as synaptic defects that can be rescued by the transgenic expression of human L1CAM, but not by the expression of the vertebrate paralogs Neurofascin and NrCAM [6]. This demonstrates a conserved role for L1CAM/Nrg from flies to humans. Finally, we found that the Nrg interaction with Semaphorin 1a has distinctive functions during guidance and synapse formation [21].

In summary, the GF circuit of Drosophila is a suitable model system for studying L1CAM function and *nrg^849^* along with *nrg^14^*;P[nrg180^ΔFIGQY^] mutants provide a unique opportunity to study pathological L1CAM missense mutations with respect to their effects on guidance and synapse formation in vivo. Here, we investigated the human L120V, E309K, H210Q, Y1070C, C264Y extracellular missense mutations in addition to a L1CAM protein mutated in the intracellular ezrin–moesin–radixin (ERM) binding site (L1-4A). The human L1 mutant proteins were expressed in both, *nrg^849^* and *nrg^14^*;P[nrg180^ΔFIGQY^] mutant backgrounds to gauge the abilities of these proteins to rescue axon guidance and synapse development defects, respectively [26].

## Materials and Methods

### Fly Stocks

The following fly stocks generated have been previously described [27]: UAS-L1-Y1070C, UAS-L1-C264Y, UAS-L1-H210Q, UAS-L1-R184Q, UAS-L1-E309K, and UAS-L1-L120V. The cDNAs for L1CAM, L1-4A and L1-1180 obtained from Vance Lemmon (University of Miami, Miller School of Medicine) were re-cloned into the pUAST vector via *Eco*RI-*Xho*I restriction sites [26] and transgenic fly lines were established on the second chromosome (Cutaneous Biology Research Center fly core, Mass. General Hospital). The P{GawB}OK307 Gal-4 driver (referred to as OK307), UAS-CD8-GFP as well as *nrg^849^* were obtained from the Bloomington Stock Center. *nrg^14^*;P(nrg180^ΔFIGQY^) was generated by the Jan Pielage lab (Friedrich Mieschner Institute, Switzerland) as previously described [20]. For the rescue experiments, a *nrg^849^*;OK307/CyO stock and a *nrg^849^*;OK307, UAS-CD8-GFP/CyO stock as well as OK307,UAS-L1/CyO stocks for all L1-constructs were established. Male flies of the UAS-L1 or UAS-L1-variants were crossed to female *nrg^849^* flies. In addition, male flies of the OK307,UAS-L1/CyO (or L1-variants) stocks were crossed to female *nrg^14^*;P(nrg180^ΔFIGQY^) flies. All genetic crosses were performed at 25°C and the male progeny of the crosses were tested when three to four days old.

### Western Blot

For the developmental Western blot, one pupa or one adult OK307/+;UAS-L1/+ fly homogenized in Laemmli buffer with Halt™ Protease and Phosphatase Inhibitor Cocktail (Thermo Scientific, 1∶100 dilution) was loaded per lane. Ten fly heads of offspring from UAS-L1 and UAS-L1-variants crossed to OK307 were used to extract protein for comparing expression between wild type L1CAM and L1CAM with pathological missense mutations. SDS-PAGE was performed according to standard protocol. The Western blots were incubated with the anti-L1 C20 antibody (SC-1508, Santa Cruz) at a dilution of 1∶200. Anti-Actin (Santa Cruz, SC-1616) was used as a loading control at a dilution of 1∶200. Secondary donkey anti-goat antibody (SC-2020, Santa Cruz) was used at a concentration of 1∶1000.

### Immunohistochemistry and Anatomical Analysis

Flies (3–5 days old) were dissected to expose the cervical connective and the ventral nerve cord as previously described [28]. The GFs were injected in the cervical connective with 10% W/V Tetramethylrhodamine-Dextran (Invitrogen) or 10 mM Alexa Flour 555 Hydrazide (Molecular Probes) by passing depolarizing or hyperpolarizing current respectively. In addition, co-expression of UAS-CD8-GFP with UAS-L1 and UAS-L1 variants was used to visualize the GFs in the brain. The immunohistochemical staining procedure for confocal microscopy has been described previously [29]. The GFP signal was enhanced with anti-GFP antibody (Invitrogen, 1∶750 dilution) and goat anti-rabbit-Cy2 (Jackson ImmunoResearch, 1∶500 dilution). For L1CAM immunostaining, intracellular anti-L1 C20 antibody (SC-1508, Santa Cruz, 1∶50 dilution) and donkey anti-goat-Cy2 (Jackson ImmunoResearch, 1∶500 dilution) were used. Samples were analyzed using a Nikon C1si Fast Spectral Confocal system using the same scan settings between different samples and the images were processed using Nikon Elements Advanced Research 4.1 software image processing software. The number of GFs that exited the brain and reached the synaptic target area were plotted in a bar graph as percent wild type for guidance defects. Statistical significance between the *nrg^849^* and the mutant L1 rescues was obtained by Chi-square analysis using Microsoft Excel (Microsoft Office 10).

### Electrophysiology

Electrophysiological recordings from the Dorsal-longitudinal Muscle (DLM) and the TTM Tergo-trochanteral Muscle (TTM) of the GF circuit were obtained as described previously [30]. Recordings from the DLM were used to determine the presence of the GF in the synaptic target area. Flies that did not exhibit a DLM response were not included in the characterization of the synaptic defects between the GF and the TTMn. The Following Frequencies were determined as the number of responses in percent of ten trains of ten stimuli at 100 Hz with a two second interval between the trains. For the Response Latencies, individual stimuli were used to determine the time delay between the stimulation of the GFs and the recording of a response in the TTM. The raw data was plotted on a scatter plot in Excel (Microsoft Office 10). Statistical significance was tested with Student’s t-test in Sigma Plot 12 software. In addition, the average Following Frequencies of all flies, the average Response Latency of all responding flies, as well as the percent of flies that did not exhibit a TTM response when stimulated in the brain were calculated using Microsoft Excel (Microsoft Office 10). Thoracic stimulation was used to directly activate the TTM motor neurons and to confirm that the observed defect was at the GF to TTMn connection and not at the neuromuscular junction, for those animals that didn’t show a TTM response with brain stimulation.

## Results

### Expression of Human Pathological L1CAM Missense Mutations in the Drosophila Nervous System

When expressed in different cell lines, several human L1CAM proteins with missense mutations in their extracellular domain are retained in the endoplasmic reticulum [13], [14], [15], [16], [17]. In order to determine in vivo expression in the Drosophila nervous system, we used the Gal4/UAS system to transgenically express wild-type human L1CAM and L1CAM with pathological extracellular missense mutations (L120V, Y1070C, C264Y, H210Q, E309K and R184Q, Fig. 1A) [15], [31]. Previously it has been shown that expression of L1CAM in Drosophila larvae resulted in a 200 kDa form [27]. In Western blots of adult flies using an antibody against the intracellular domain of human L1CAM, wild-type human L1CAM was detected as a 200 kDa band in addition to a more prominent 65 kDa band (Fig. 1B). This suggests L1CAM is proteolytically cleaved in adult Drosophila and therefore we determined its cleavage developmentally in pupal stages, as well as in younger (1 day) and older (8 days) adults. During early pupal development the majority of L1CAM can be detected as a 200 kDa band (Fig. 1B). However, as development proceeds the overall amount of L1CAM remained approximately the same, but the ratio of cleaved L1CAM to full length L1CAM increased with maximum cleavage occurring in the adult (Fig. 1B). In contrast, no difference was seen between one and eight day old adult flies. These results were consistently observed in five independent Western blots. Thus, L1CAM cleavage seems to correlate with the maturation of the nervous system and is dependent on the differentiation status of neurons in the nervous system. Transgenic expression of all tested L1CAM constructs with pathological missense mutations resulted in robust expression in the adult (Fig. 1B). The 200 kDa form was detected with equal or higher strength when compared to wild-type L1CAM. With the exception of L1-C264Y, the proteolytically cleaved 65 kDa L1CAM product was observed for all human L1CAM proteins with extracellular missense mutations (Fig. 1C).

### Testing the Abilities of Human Mutant L1CAM Proteins to Rescue GF Axonal Guidance Defects in *nrg^849^* Mutant Background

We previously showed that the extracellular missense mutation in the second Ig domain of *nrg^849^* mutants (Fig. 1A) results in GF guidance defects in the brain [6]. These defects can be rescued by the transgenic expression of human L1CAM in the GF [6], [23].

**Figure 2:**
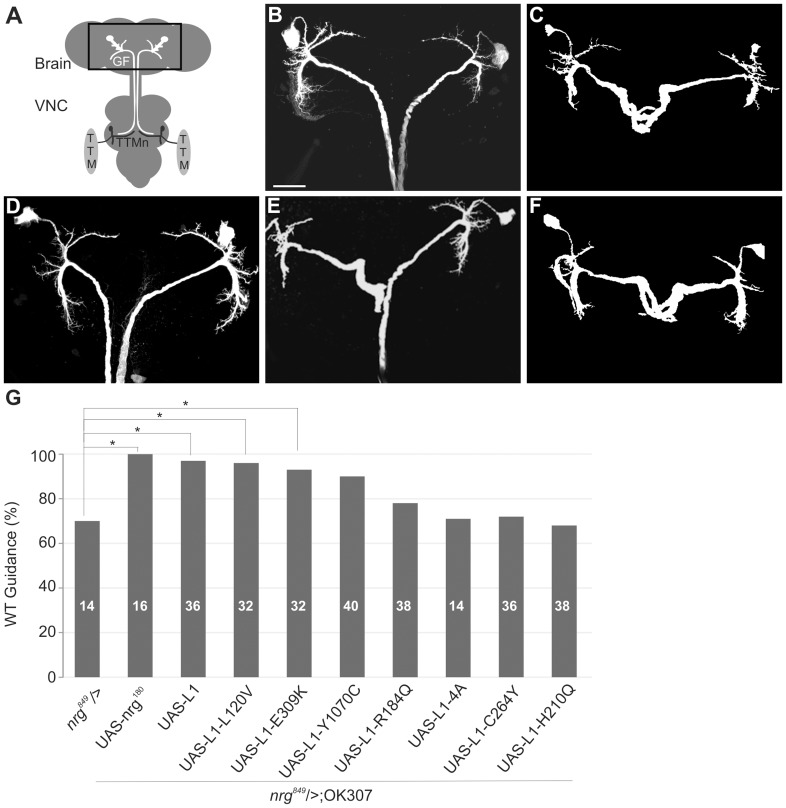
Anatomical characterization of GF guidance defects of mutant L1CAM protein expressions in *nrg^849^* background. (A) Schematic of the giant fiber (GF) to TTM (Tergo-trochanteral Muscle) circuit in Drosophila. The GF soma in the brain extends an axon into the second neuromere of the VNC, where it synapses with the Tergo-trochanteral motor neuron (TTMn), which itself innervates the TTM. Rectangle highlights the brain region depicted in B-F. (B) GF anatomy in the brain of a wild-type control fly. Scale bar represents 50 µm. (C) In a *nrg^849^* animal two GFs were seen to stall in the suboesophageal ganglion. (D) Expression of wild-type human L1CAM in the GF in the *nrg^849^* background rescued the axonal guidance defect. Expression of L1-Y1070C (E) or L1-H210Q (F) in the *nrg^849^* background did not rescue or only partially rescued the axonal guidance phenotype. (G) Bar graph shows the quantification of the axonal guidance defects. The rescue of the *nrg^849^* guidance phenotype by expression of UAS-nrg^180^, UAS-L1 and the various mutant L1-constructs represented in percent values. The significant differences (Chi-square analysis, p≤0.05) between Nrg^180^, L1, L1-L120V and L1-E309K expression in the *nrg^849^* background and negative control flies (*nrg^849^*) are indicated by asterisks.

In wild-type flies each GF cell body in the brain projected an axon through the cervical connective into the second neuromere of the ventral nerve cord (VNC) and made synaptic connections with the TTMn motor neurons (Fig. 2 A, B). In *nrg^849^* mutants one or both GFs were stalled in the suboesophageal ganglion of the brain with a penetrance of approximately 30% (Fig. 2C, G), while the remaining GFs, once they exited the brain, continued to grow to the synaptic target area. Expression of UAS-L1 with the OK307 Gal-4 driver in the GF of *nrg^849^* animals significantly rescued the guidance defects in virtually all specimens (Fig. 2D, G). We also expressed human L1CAM with the six extracellular pathological missense mutations (Fig. 1 A, L120V, Y1070C, C264Y, H210Q, E309K and R184Q) in the *nrg^849^* mutant background to test their ability to complement for the loss of homophilic binding of the Nrg^849^ protein and to rescue the guidance defects in vivo. In addition, we also expressed UAS-L1-4A in which four amino acids in the juxtamembrane region (KGGKYSV, Fig. 1A) were substituted with alanine residues [26]. This ERM-binding site plays an important role in axon branching, but is not required for neurite outgrowth [26]. To reveal the anatomical guidance phenotypes in the brain, we co-expressed UAS-CD8-GFP as a reporter with the L1-constructs. Additionally, we determined the number of GFs exiting the brain in animals not expressing CD8-GFP by using differential interference contrast (DIC) microscopy to identify the GFs in the cervical connective. Subsequent dye-injections of the GFs in the cervical connective were performed to confirm that all GFs that exited the brain reached the synaptic target area in the second neuromere of the VNC. We found that Nrg^180^, L1CAM, and L1CAM with L120V and E309K mutations were able to significantly rescue the guidance defects (p<0.05, Chi-square statistical analysis) in *nrg^849^* animals. In contrast, the expression of L1-C264Y, L1-H210Q, L1-R184Q and L1-4A did not complement the loss of homophilic binding in *nrg^849^* animals. Although *nrg^849^* animals expressing L1-Y1070C were statistically insignificant from negative control animals (*nrg^849^*), they were also statistically insignificant from positive control animals (L1CAM in *nrg^849^* background). Therefore, the observed reduction in the guidance defects suggests that expression of L1-Y1070C is able to partially rescue guidance defects in vivo.

### Determining the Ability of Human Pathogenic L1CAM Mutations to Rescue Synaptic Defects in *nrg^14^*;P[nrg180^ΔFIGQY^] Mutant Background

**Figure 3:**
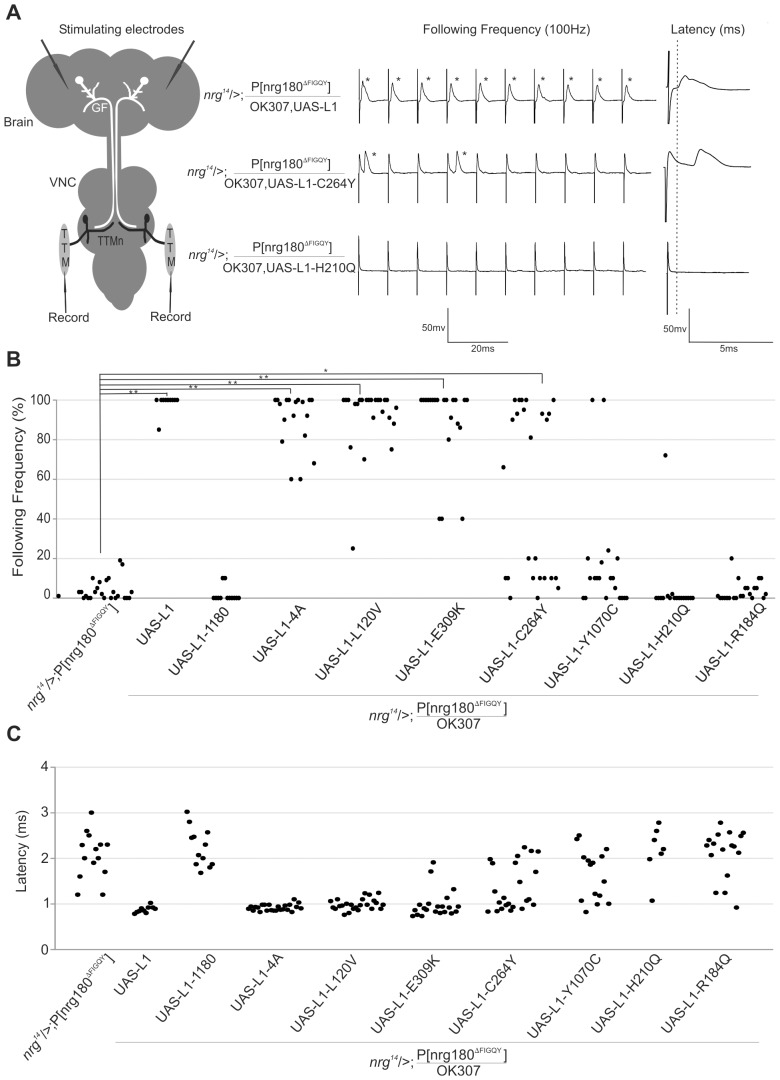
Functional characterization of GF-TTMn synaptic defects of mutant L1CAM protein expressions in *nrg^14^*;P[nrg180^ΔFIGQY^] background. (A) Schematic of the GF to TTM circuit in Drosophila. For electrophysiological recordings, tungsten electrodes were inserted into the brain for the GF stimulation. The output of the neuronal circuit was recorded from the TTM muscles with glass electrodes. Sample electrophysiological recordings from GF-TTM pathway. In positive control animals (*nrg^14^*/>;P[nrg180^ΔFIGQY^]/OK307,UAS-L1) the GF-TTM pathway was able to follow reliably at a one-to-one ratio when the GFs were stimulated in the brain with ten stimuli given at 100 Hz and the Response Latency was 0.87 ms (dashed grey line). In animals, which express L1-C264Y and L1-H210Q protein in a *nrg^14^*/>;P[nrg180^ΔFIGQY^] background, only few or no responses (asterisks) could be recorded when the GFs were stimulated in the brain. The Response Latency of all responses was increased. (B) Scatter plots of Following Frequencies. Following Frequencies were only significantly increased (** = p<0.001, * = p value ≤0.05, Mann-Whitney Rank Sum Test) in *nrg^14^*;P[nrg180^ΔFIGQY^] in animals that expressed UAS-L1, UAS-L1-E309K, UAS-L1-L120V and UAS-L1-4A driven by OK307. (C) Scatter plots of Response Latencies. In some animals no responses could be recorded from the TTM when the GF was stimulated in the brain (see Table 1) and thus were not included in the scatter plot. However, in most responding animals that had a decreased ability to follow at a one-to-one ratio at 100 Hz, the Response Latency was increased indicating a reduction in synaptic strength for the GF to TTMn connection.

**Figure 4:**
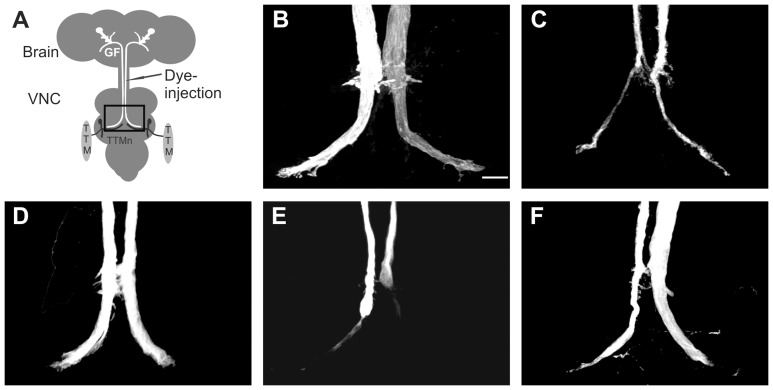
Anatomical characterization of GF-TTMn synaptic defects of L1CAM constructs in *nrg^14^*;P[nrg180^ΔFIGQY^] background. (A) Schematic of the GF to TTM circuit in Drosophila. Rhodamine-dextran was injected into the GF in the cervical connective to visualize the synaptic terminal. Rectangle highlights the region in the thoracic ganglion depicted in B-F. (B) Wild-type control flies exhibited a large synaptic terminal in the second neuromere of the VNC. Scale bar represents 10 µm. (C) The GF terminals of *nrg^14^*;P[nrg180^ΔFIGQY^] were skinnier (and sometimes also shorter) when compared to wild-type control flies. (D) The size of the synaptic terminal was restored in *nrg^14^*;P[nrg180^ΔFIGQY^] animals that expressed UAS-L1 driven by OK307. (E) The synaptic terminal in *nrg^14^/>*;P[nrg180^ΔFIGQY^]/OK307,UAS-L1-Y1070C animals was skinny or absent. (F) The morphology of the GF synaptic terminals of *nrg^14^*;P[nrg180^ΔFIGQY^] animals was improved by the expression of UAS-L1-4A with the OK307 Gal4-driver.

Mutations in the highly conserved intracellular FIGQY-motif of the sole L1CAM homolog Neuroglian do not affect neurite outgrowth or guidance in the GF circuit or at the larval neuromuscular junction [20]. However, in *nrg^14^*;P[nrg180^ΔFIGQY^] animals, in which the lethality of a *nrg* null mutant (*nrg^14^*) was rescued by a genomic construct in which the neuronal Nrg^180^ isoform lacks the Ankyrin-binding motif (Fig. 1A), only synaptic defects are seen in the GF circuit [20]. The synaptic GF to TTMn connection in all *nrg^14^*;P[nrg180^ΔFIGQY^] animals was severely weakened. The inability of the GF synapse to follow multiple stimuli given at 100 Hz in a one-to-one ratio (Fig. 3A, B) was associated with an increase of the Response Latency (Fig. 3A,C) and with morphologically defective synaptic terminals that were reduced in size (Fig. 4C). We have previously shown that pre- and postsynaptic expression of UAS-nrg^180^ in the GF circuit using the OK307 Gal4-driver can fully rescue the anatomical and functional synaptic phenotypes of *nrg^14^*;P[nrg180^ΔFIGQY^] mutant animals [20]. Here we used expression of human L1CAM and expression of L1-1180, which lacks part of the intracellular domain including the FIGQY motif [14], as positive and negative controls for the effects of expression of human pathogenic L1CAM mutations. We found that transgenic expression of human L1CAM efficiently rescued the synaptic defects in *nrg^14^*;P[nrg180^ΔFIGQY^] mutants (Fig. 3, Table 1). The synaptic function was significantly improved in all animals. The average Following Frequency increased from 4% to 98.5%, the average Response Latency decreased from 2.05 ms to 0.87 ms in *nrg^14^*;P[nrg180^ΔFIGQY^] and the number of animals with no TTM responses upon GF stimulation in the brain decreased from 37% to 0% in animals that express human L1CAM (Table 1). In addition, the morphology of the synaptic terminals was restored (Fig. 4D). In contrast, in the negative control animals (*nrg^14^*;P[nrg180^ΔFIGQY^]; OK307, UAS-L1-1180), no improvement of the synaptic phenotypes was observed (Fig. 3, Table 1). Thus *nrg^14^*;P[nrg180^ΔFIGQY^] mutants enabled us to test whether human pathological L1CAM mutations can rescue the synaptic phenotypes or whether the extracellular missense mutations cannot compensate for the lack of outside-in signaling via the FIGQY motif.

**Table 1:** Electrophysiological phenotypes of L1CAM-constructs in *nrg^14^*;P[nrg180^ΔFIGQY^] background.

Genotype	n	FF in %	NR in%	RL in ms
OK307,UAS-L1	16	99.75±0.17	0	0.87±0.02
OK307,UAS-L1-L1180	16	99.3±0.63	0	0.9±0.02
OK307,UAS-L1-4A	20	99.5±0.05	0	0.84±0.01
OK307,UAS-L1-L120V	20	99.5±4.5	0	0.78±0.04
OK307,UAS-L1-E309K	12	99.5±0.26	0	0.9±0.16
OK307,UAS-L1-C264Y	25	100±0	0	0.9±0.03
OK307,UAS-L1-Y1070C	20	99.2±2.46	0	0.9±0.002
OK307,UAS-L1-H210Q	22	99.2±1.89	0	1.02±0.03
OK307,UAS-L1-R184Q	18	99.6±0.33	0	0.8±0.02
*nrg^14^*/>;P[nrg180^ΔFIGQY^]	24	4.04±1.10	37	2.05±0.13
*nrg^14^*/>;P[nrg180^ΔFIGQY^]/OK307,UAS-L1	10	98.5±1.5	0	0.87±0.02
*nrg^14^*/>;P[nrg180^ΔFIGQY^]/OK307,UAS-L1-1180	12	2±1.12	83	2.24±0.12
*nrg^14^*/>;P[nrg180^ΔFIGQY^]/OK307,UAS-L1-4A	18	90±3.33	0	0.91±0.01
*nrg^14^*/>;P[nrg180^ΔFIGQY^]/OK307,UAS-L1-L120V	24	92±3.42	0	0.98±0.02
*nrg^14^*/>;P[nrg180^ΔFIGQY^]/OK307,UAS-L1-E309K	21	89±4.62	0	0.98±0.07
*nrg^14^*/>;P[nrg180^ΔFIGQY^]/OK307,UAS-L1-C264Y	25	52±8.59	8	1.36±0.11
*nrg^14^*/>;P[nrg180^ΔFIGQY^]/OK307,UAS-L1-Y1070C	20	17±6.55	25	1.64±0.13
*nrg^14^*/>;P[nrg180^ΔFIGQY^]/OK307,UAS-L1-H210Q	17	4±17	76	2.21±0.21
*nrg^14^*/>;P[nrg180^ΔFIGQY^]/OK307,UAS-L1-R184Q	22	3.5±1.08	23	2.10±0.13

Intracellular recordings from the TTM upon GF stimulation in the brain. The average Following Frequency (FF), and Response Latency (RL) shown with standard error of the mean. The TTM recordings that showed no responses (NR) upon GF stimulation in brain are shown in %. Non-responders were not included in the calculation of the average Response Latency.

Expression of L1-Y1070C, L1-H210Q and L1-R184Q failed to restore the GF synapse function (Fig. 3, Table 1), as well as the GF terminal morphology (Fig. 4E). In contrast, expression of L1-L120V, L1-E309K and L1-C264Y significantly improved the functional synaptic defects (Fig. 3 and Table 1) and morphology (data not shown) in all animals suggesting that outside-in signaling via the FIGQY motif was not disrupted by these mutations. Interestingly, L1-4A expression, unable to rescue the guidance defects of *nrg^849^* mutants, was able to significantly rescue the synaptic phenotypes of *nrg^14^*;P[nrg180^ΔFIGQY^] mutants (Fig. 3, Fig. 4F and Table 1) suggesting that the ERM binding site is more critical to GF guidance than for synapse formation.

**Figure 5:**
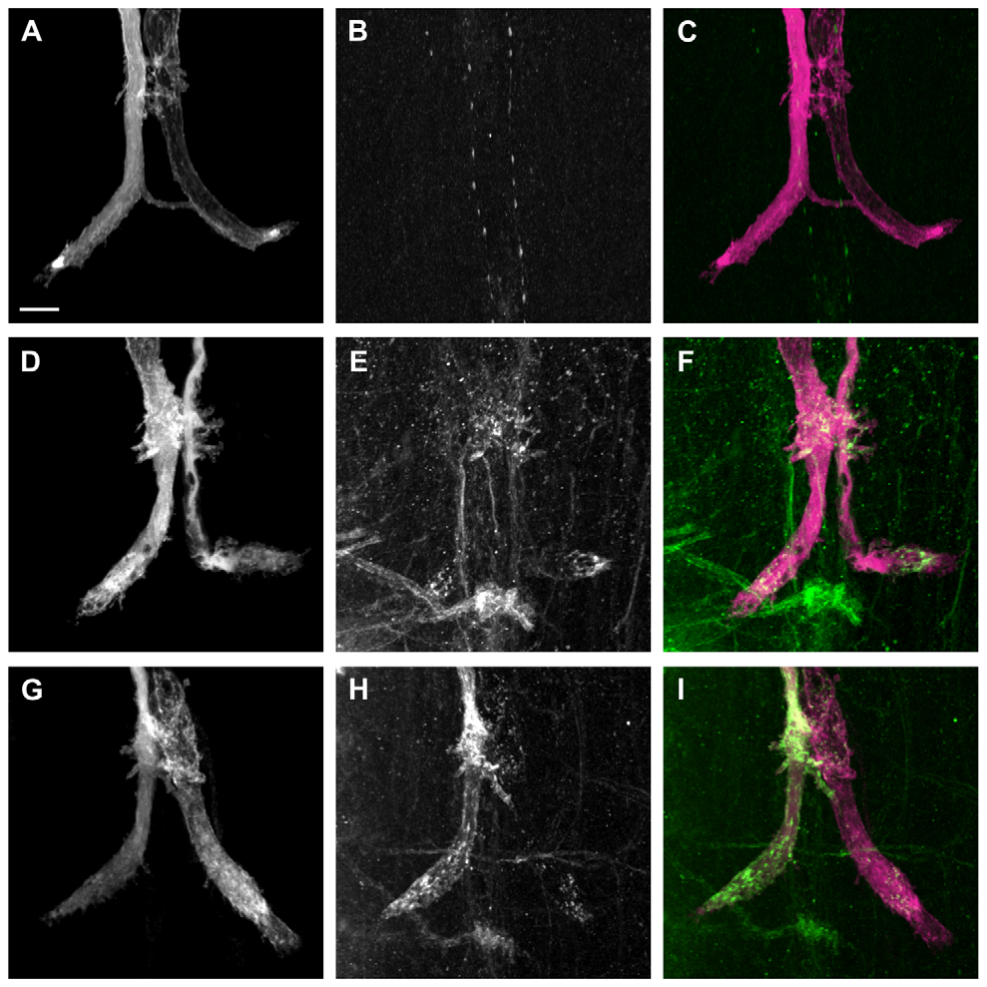
Immunohistochemical labeling of L1-C264Y at GF terminals. Alexa Fluor 555 dye injections were performed to label the GFs (magenta) and anti-L1 antibody staining is shown in green. Grayscale is used for single staining while the composite images are in corresponding colors. All images are maximum intensity projections from confocal image stacks. Scale bar is 10 µm. (A-C) OK307/+ negative control animal. The fluorescently labeled GFs (A and C, grayscale and magenta) and the ventral nerve cord (VNC) labeled with anti-L1CAM antibody (B and C, grayscale and green). Composite image is shown (C). (D–F) OK307,UAS-L1-C264Y/+ animal. The GFs were labeled with Alexa Fluor 555 (D and F, grayscale and magenta) and the VNC with anti-L1CAM (E and F, grayscale and green). Co-localization of both labels is shown in (F). L1-C264Y protein was detected in both GF terminals at the terminal surface as well as in vesicular clusters in the cytoplasm. (G–I) OK307,UAS-L1-C264Y/+ animal. The overlap of the GFs (G and I, grayscale and magenta) and anti-L1CAM staining in VNC (H and I, grayscale and green) are shown as a composite in (I). In the left GF terminal, L1-C264Y protein was strongly detected at the terminal surface as well as in the cytoplasm. In the right GF terminal, L1-C264Y protein was only labeled weakly in few vesicular clusters inside the axon terminal.

Although the average Following Frequency and average Response Latency were significantly improved with the expression of L1-C264Y (Fig. 3, Table 1), it is noteworthy that half of the tested GF to TTM connections were functionally normal, while the other half were functionally impaired like *nrg^14^*;P[nrg180^ΔFIGQY^] animals (Fig. 3B). In order to address this bimodal distribution, we immunohistochemically labeled L1CAM in OK307, UAS-L1-C264Y/+ animals (Fig. 5). We found that L1-C264Y protein (Fig. 5, green) did localize to the synaptic terminals (Fig. 5, magenta) and was seen inside as well as on the surface (Fig. 5D–I) but not in negative control animals (Fig. 5A–C). However, interestingly in some preparations the expression levels and localization of L1-C264Y protein was different for the individual GFs in the same specimen (Fig. 5G–I). While in one GF L1-C264Y protein was strongly labeled in vesicular clusters inside the GF as well as at the terminal surface (Fig. 5H, I, left GF), in the other GF only few clusters inside the terminal were detected (Fig. 5H, I, right GF). These results suggest that the different protein levels of L1-C264Y at the GF terminals are likely to be the reason that the GF-TTMn synaptic connections were rescued in some but not all *nrg^14^*;P[nrg180^ΔFIGQY^] mutant terminals.

Finally, in order to test if the expression of L1CAM with pathogenic mutations in Drosophila has any dominant negative or poisonous effects in the GF circuit, we determined putative functional defects in a wild type background (Table 1). All animals that expressed pathological L1CAM constructs with the OK307 Gal4 driver developed a GF synapse that was functionally similar to animals that expressed wild-type L1CAM (Table 1). This suggests that the failure of pathological L1CAM constructs to rescue guidance or synaptic phenotypes of *nrg^849^* or *nrg^14^*;P[nrg180^ΔFIGQY^] mutants is due to the loss of a biological function and not due to a dominant negative or poisonous effect.

## Discussion

### Expression and Proteolytic Cleavage of Mutant Human L1CAM Proteins

Many different pathological L1CAM mutations have been characterized for their effects on homophilic and heterophilic interactions, as well as on their cell surface expression levels in vitro. However, there are only a few studies on their biological consequences in vivo [15], [32], [33]. Here we characterized extra- and intracellular human L1CAM mutations in vivo with respect to expression along with their ability to support axon guidance and synapse formation in the GF circuit of Drosophila.

In vertebrates, L1CAM is proteolytically cleaved by various enzymes, such as neuropsin, plasmin, ADAM10/17 and a yet unidentified serine protease [9], [34], [35], [36], [37]. We found that expression of L1CAM in the Drosophila nervous system resulted in proteolytic cleavage to a 65 kDa fragment detected by antibodies directed against the intracellular L1CAM domain (Fig. 1B, C). It is important to note that this proteolytic cleavage was not seen in larvae but in adults, suggesting that this cleavage is dependent on the differentiation status of the nervous system [27]. We found that the overall ratio of cleaved L1CAM to full-length L1CAM increased from pupa to adult and the cleavage rate remained constant when comparing adults of different ages. However, it remains to be determined if the cleavage in Drosophila occurs at a site homologous to those that have been described in vertebrates and if this particular cleavage is of functional relevance. Although, proteolytic cleavage of Nrg has not yet been described, we have evidence that it does get cleaved (unpublished data, manuscript in preparation). We found that transgenically expressed L1CAM proteins with L120V, Y1070C, H210Q, E309K and R184Q mutations were proteolytically cleaved and had similar or higher expression levels as the wild-type L1CAM-construct. This allowed for a functional analysis of the pathogenic L1CAM mutant proteins in terms of their ability to support axonal guidance and synapse formation in Drosophila mutants.

The overall L1-C264Y expression was reduced comparatively but in Western blots no cleavage was observed for L1-C264Y protein even with ten heads (Fig. 1C). This is similar to L1-C264Y expression in the vertebrates. In vitro the C264Y mutation leads to reduced cell surface expression of the mutant protein, while in vivo L1-C264Y protein seems to be absent or below detection threshold at the cell surface [15], [33], [38], [39]. Therefore, the absence of L1-C264Y cleavage in the vertebrate nervous system was associated with its lack of cell surface expression [39]. However, expression of L1-C264Y in Drosophila was able to fully rescue the synaptic defects of half of the GF-TTMn connections in *nrg^14^*;P[nrg180^ΔFIGQY^] animals suggesting that L1-C264Y protein was expressed at the cell surface. Correlating with the electrophysiological data, we did find that transgenically expressed L1-C264Y protein was present in numerous though not all synaptic terminals at the cell surface as well as in vesicular clusters inside the terminal. Therefore, our results suggest that in the Drosophila GF the pathogenic C264Y mutation not only affects proteolytic processing indirectly by reducing cell surface expression but may also affect it directly in L1-C264Y protein that is expressed at the cell surface.

### L1CAM Mutations in Axon Guidance

Homophilic L1-L1 interactions mediated by the extracellular Ig-domains are involved in various L1-dependent signaling processes required for axonal growth, guidance and synapse stability [3], [6], [20]. The S213L mutation in the *nrg^849^* allele is at a site analogous to the position of the H210Q mutation in humans and both mutations affect homophilic L1 binding [23], [32] (Fig. 1A). However, although L1CAM-H210Q proteins are unable to interact homophilically with each other, they can efficiently bind to wild-type L1CAM [40]. We have shown that Neuroglian is required pre- and postsynaptically for GF synapse formation and that expression of Nrg^180^ on either side of *nrg^849^* mutant synapses can partially rescue the synaptic phenotypes. This suggests that Nrg-S213L similar to L1-H210Q is able to bind to a wild-type extracellular domain [6], [20], [21].

Here, we tested whether the expression of different pathological mutations can compensate for the loss of homophilic interaction in *nrg^849^* mutants. Expression of wild-type human L1CAM, as well as L1-L120V and L1-E309K, both of which mediate normal homophilic binding, was able to efficiently rescue the guidance defects of *nrg^849^* mutants (Fig. 2) [15], [32], [33]. However, previously it was shown that expression of L1-E309K in a null mutant background did not rescue the guidance defects of bristle mechanosensory (BM) neurons [27]. This suggests that either the functional Nrg/L1CAM requirements for guidance in BM and GF neurons are different or that homophilic interaction of L1-E309K protein with Nrg^849^ protein induces signaling that is sufficient for GF guidance.

In contrast, several L1-CAM constructs failed to rescue the guidance phenotypes. This may be due to one or a combination of three most likely causes. First, L1CAM missense mutations that severely reduce or prevent cell surface expression of the mutant protein are likely to affect the rescue capacity, e.g. the C264Y mutation [15], [33], [38], [39]. Secondly, L1CAM missense mutations that affect homophilic binding are unable to fully complement for the lack of homophilic binding of the Nrg-S213L protein. This appears to be the case for the H210Q, C264Y and R184Q mutations (Fig. 2) [15], [32]. Finally, it is conceivable that some pathological missense mutations with normal homophilic binding have impaired outside-in signaling or heterophilic interactions that are required for axon guidance [27]. These mutant proteins are also unlikely to rescue the *nrg^849^* guidance phenotype. Although the L1-Y1070C construct showed a strong improvement in restoring *nrg^849^* guidance defects (Fig. 2), despite its normal homophilic binding capacity, it did not reach statistical significance. However, this mutation has been shown to reduce Epidermal Growth Factor Receptor (EGFR) signaling critical for axon growth in Drosophila [27]. In addition, we found that expression of L1-4A failed to rescue the *nrg^849^* guidance phenotypes (Fig. 2). The ERM-binding site has been shown to be critical for axon growth and branching in vertebrates [26], [41], [42], suggesting it is also required for axon guidance in Drosophila.

### L1CAM Mutations in Synapse Formation

While many pathological L1CAM mutations have been studied for their effects on neurite outgrowth, axon guidance and branching, virtually no information is available with respect to their impact on synapse development. We have previously demonstrated that intracellular signaling of the Nrg^180^ FIGQY motif is essential for synapse formation of the GF, but not for neurite outgrowth or axon guidance [20]. Our published results demonstrate that expression of wild-type Nrg^180^ on either side of Nrg^ΔFIGQY^ synaptic terminals is able to rescue the morphological and functional defects. This finding suggests that transcellular Nrg^180^ signaling can superimpose the molecular information to the other synaptic side, which lacks Nrg-FIGQY signaling. However, it remains to be determined if this transcellular interaction is homophilic, heterophilic or both.

The finding that human L1CAM mutations like L1-L120V, L1-E309K, L1-4A and partially L1-C264Y were able to rescue the synaptic defects of Drosophila *nrg^14^*;P[nrg180^ΔFIGQY^] mutant animals (Fig. 3, 4) suggests that they do not affect outside-in signaling via the FIGQY motif. However, the results do not exclude a synaptic function via other signaling mechanisms that are independent of the FIGQY motif and are unaffected in the Nrg180^ΔFIGQY^ protein. We identified several L1CAM mutations that had expression levels (Fig. 1C) comparable to L1CAM wild-type control flies, but did not rescue the synaptic phenotype. Although L1-H210Q protein is able to bind to wild-type L1CAM [40], both human L1CAM mutations with disrupted homophilic binding (L1-H210Q and L1-R184Q) were unable to rescue the synaptic defects when expressed pre- and postsynaptically in the GF circuit of *nrg^14^*;P[nrg180^ΔFIGQY^] mutant flies. This suggests that L1-H210Q and L1-R184Q may not interact transcellularly with Nrg^ΔFIGQY^ and homophilic interactions are also essential for synapse formation. Alternatively, L1-H210Q and L1-R184Q may be able to interact transcellularly with Nrg^ΔFIGQY^, which has a wild-type extracellular domain. However, in this scenario the H210Q and R184Q mutations disrupt outside-in signaling processes via the FIGQY motif. The latter hypothesis is supported by our finding that the *nrg^849^* mutants with the analogous L1-H210Q mutation have reduced tyrosine phosphorylation of the FIGQY motif and synaptic phenotypes similar to *nrg^14^*;P[nrg180^ΔFIGQY^] mutants in addition to guidance defects [6]. Finally, we found that L1-Y1070C mutant protein failed to rescue the synaptic defects of *nrg^14^*;P[nrg180^ΔFIGQY^] mutant flies although its homophilic interactions are not affected by the mutation [15], [32]. Both, Y1070C and E309K mutations have been shown to reduce EGFR signaling to a similar extent [27]. However, because the L1-Y1070C transgenic expression level was higher than the expression of L1-E309K (Fig. 1 C), the lack of rescue capacity of the L1-Y1070C protein is unlikely to be due to reduced EGFR signaling. This suggests that the Y1070C mutation either affects FIGQY phosphorylation or the localization of phosphorylated and non-phosphorylated L1-type protein at the cell surface. Phosphorylated and non-phosphorylated L1-type proteins have been shown to localize to distinct areas [43] and interestingly, heterophilic binding to Tag-1/Axonin-1 and contactin/F11 are increased for the L1-Y1070C mutant protein [15], [32].

In summary, we find that extracellular human pathogenic L1CAM missense mutations not only affect adhesive properties but also intracellular signaling pathways distinctly, which are required for axon guidance, synapse formation or both. Mutations that affect homophilic binding are most detrimental because they affect adhesive properties but often also heterophilic interactions and intracellular signaling [6], [13], [15], [27]. In contrast, intracellular mutations as well as extracellular mutations that only affect heterophilic interactions are more likely to only result in a partial loss of L1CAM biological function, which is also reflected by the fact that pathogenic intracellular missense mutations are known to result in less detrimental pathological phenotypes [13]. Therefore, these types of mutations may affect different biological processes such as guidance and synapse formation distinctively and their characterization in vivo is essential in order to gain a complete understanding of L1CAM function. We find that outside-in signaling via the ERM-motif and via the FIGQY motif are required for GF guidance and synapse formation in Drosophila, respectively. Here, we provided novel evidence that the H210Q, R184Q and Y1070C but not the L120V and E309K L1CAM mutations affect outside-in signaling via the Ankyrin binding domain, which is essential for synapse formation but not for axon guidance. Thus, the broad variability of pathological phenotypes observed between humans with L1CAM mutations is based on the differential effects on distinct signaling pathways required for developmental biological processes.
